# Impact of pitavastatin on new-onset diabetes mellitus compared to atorvastatin and rosuvastatin: a distributed network analysis of 10 real-world databases

**DOI:** 10.1186/s12933-022-01524-6

**Published:** 2022-05-23

**Authors:** Won-Woo Seo, Seung In Seo, Yerim Kim, Jong Jin Yoo, Woon Geon Shin, Jinseob Kim, Seng Chan You, Rae Woong Park, Young Min Park, Kyung-Jin Kim, Sang Youl Rhee, Meeyoung Park, Eun-Sun Jin, Sung Eun Kim

**Affiliations:** 1 Departments of Internal Medicine, Kangdong Sacred Heart Hospital, Hallym University College of Medicine, 150, Seongan-ro, Gangdong-gu, Seoul, 05355 South Korea; 2 Institute for Liver and Digestive Diseases, Hallym University, Chuncheon, South Korea; 3 Departments of Neurology, Kangdong Sacred Heart Hospital, Hallym University College of Medicine, Seoul, South Korea; 4 Department of Epidemiology, School of Public Health, Seoul National University, Seoul, South Korea; 5 Department of Biomedical Systems Informatics, Yonsei University College of Medicine, Seoul, South Korea; 6 Department of Biomedical Informatics, Ajou University, Suwon, South Korea; 7 Department of Family Medicine, National Health Insurance Service Ilsan Hospital, Goyang, South Korea; 8 Department of Internal Medicine, Ewha Womans University Medical Center, Ewha Womans University School of Medicine, Seoul, South Korea; 9 Department of Endocrinology and Metabolism, Kyung Hee University School of Medicine, Seoul, South Korea; 10 Biomedical Research Institute, Pusan National University Hospital, Busan, South Korea; 11 Cardiovascular Center, Kyung Hee University Hospital at Gangdong, Kyung Hee University, Seoul, South Korea

**Keywords:** Diabetes mellitus, Pitavastatin, Statin, Common data model

## Abstract

**Background:**

Statin treatment increases the risk of new-onset diabetes mellitus (NODM); however, data directly comparing the risk of NODM among individual statins is limited. We compared the risk of NODM between patients using pitavastatin and atorvastatin or rosuvastatin using reliable, large-scale data.

**Methods:**

Data of electronic health records from ten hospitals converted to the Observational Medical Outcomes Partnership Common Data Model (n = 14,605,368 patients) were used to identify new users of pitavastatin, atorvastatin, or rosuvastatin (atorvastatin + rosuvastatin) for ≥ 180 days without a previous history of diabetes or HbA1c level ≥ 5.7%. We conducted a cohort study using Cox regression analysis to examine the hazard ratio (HR) of NODM after propensity score matching (PSM) and then performed an aggregate meta-analysis of the HR.

**Results:**

After 1:2 PSM, 10,238 new pitavastatin users (15,998 person-years of follow-up) and 18,605 atorvastatin + rosuvastatin users (33,477 person-years of follow-up) were pooled from 10 databases. The meta-analysis of the HRs demonstrated that pitavastatin resulted in a significantly reduced risk of NODM than atorvastatin + rosuvastatin (HR 0.72; 95% CI 0.59–0.87). In sub-analysis, pitavastatin was associated with a lower risk of NODM than atorvastatin or rosuvastatin after 1:1 PSM (HR 0.69; CI 0.54–0.88 and HR 0.74; CI 0.55–0.99, respectively). A consistently low risk of NODM in pitavastatin users was observed when compared with low-to-moderate-intensity atorvastatin + rosuvastatin users (HR 0.78; CI 0.62–0.98).

**Conclusions:**

In this retrospective, multicenter active-comparator, new-user, cohort study, pitavastatin reduced the risk of NODM compared with atorvastatin or rosuvastatin.

**Supplementary Information:**

The online version contains supplementary material available at 10.1186/s12933-022-01524-6.

## Background

Statins reduce blood cholesterol levels and are widely used for the primary or secondary prevention of cardiovascular diseases [1, 2]. Although statin treatment is generally considered safe, several recent studies have suggested that it confers an increased risk of new-onset diabetes mellitus (NODM) [3, 4]. Overall, statins have been found to increase the risk of NODM by 10–12%, and the risk is slightly greater with high-intensity statin therapy than with low- or moderate-intensity therapy [4]. However, due to the limited data directly comparing statins, the risk of NODM among individual statins remains controversial. A previous meta-analysis showed no significant difference in diabetic risk among various statins, while a population-based, retrospective, cohort study showed that atorvastatin and simvastatin were associated with an increased risk of NODM compared to pravastatin [3, 5, 6]. Moreover, Yoon et al. reported that the atorvastatin-exposed cohort had the highest risk of NODM compared with the matched, non-exposed cohort among various statins [7]. In contrast, a meta-analysis of 17 studies reported that rosuvastatin was associated with the highest risk of NODM among various statins [8].

Pitavastatin is a highly potent statin along with atorvastatin and rosuvastatin. LDL-C decreased by an average of 47% after treatment with pitavastatin 4 mg, which was comparable to atorvastatin 40 mg or rosuvastatin 10 mg [9]. Previous randomized clinical trial demonstrated that pitavastatin 4 mg compared with pitavastatin 1 mg therapy significantly reduced LDL-C and clinical outcomes irrespective of renal function state [10]. When used in combination, pitavastatin and pemafibrate improved lipid profile and endothelial function in hypertension and insulin resistance model rats [11]. Ihm et al. also reported combination therapy with pitavastatin and fenofibrate more effectively reduced non-HDL-C compared with pitavastatin monotherapy in patients with a high risk for cardiovascular disease [12]. Several previous studies have reported that pitavastatin had less influence on the development of diabetes mellitus or glucose metabolism than other statins, such as pravastatin, atorvastatin, or rosuvastatin [13–16]. Additionally, pitavastatin tended to have a slightly lower hazard ratio (HR) for NODM than other statins in a real-world cohort study of Asian patients [7]. In contrast, a single-center, retrospective study including 3680 patients reported that pitavastatin was more strongly associated with NODM than other statins [17]. These inconclusive results might be due to limited data for direct comparisons of individual statins and various biases, including selection, immortal, protopathic, and/or confounding bias originating from research design error, relatively small sample size, or analysis method.

In present new-user model cohort study, we assessed the impact of pitavastatin on NODM compared with atorvastatin or rosuvastatin, the most commonly prescribed statins in the world, in patients without diabetes or impaired glucose tolerance using the Observational Medical Outcomes Partnership (OMOP)- Common Data Model (CDM) of large-scale data validated in our previous studies [18–20].

## Methods

### Data sources

This multicenter, controlled cohort study included real-world clinical data of 14,605,368 patients from 10 secondary or tertiary hospitals in Korea converted to the OMOP-CDM version 5.3. The breakdown was as follows: (1) Kangdong Sacred Heart Hospital CDM (KDH; 1,689,604 patients); (2) Kyung Hee University Hospital at Gangdong CDM (KHNMC; 822,183 patients); (3) Wonkwang University Hospital CDM (WKUH; 1,001,794 patients); (4) Daegu Catholic University Medical Center CDM (DCMC; 1,688,980 patients); (5) Ajou University Medical Center CDM (AUMC; 3,109,677 patients); (6) Pusan National University Hospital (PNUH; 1,753,001 patients); (7) Ewha Womans University Mokdong Hospital CDM (EUMC; 1,745,549 patients); (8) National Health Insurance Service Ilsan Hospital CDM (NHIMC; 1,367,483 patients); (9) Myongji Hospital (MJH; 882,646 patients); and (10) Kangwon National University Hospital CDM (KWMC; 544,451 patients). All databases comprise de-identified, patient-level, electronic health record data converted into the standard vocabulary of the CDM to generate network-wide results through distributed research networks using the same analysis program among collaborating organizations [21, 22].

### Study design

We conducted a multicenter, retrospective, observational, comparative, new-user cohort study. Patients aged ≥ 18 years who were first exposed to pitavastatin, atorvastatin, or rosuvastatin were included in this study. For consistency in the definition of “new-users” to minimize immortal-time bias, we only included patients who had a continuous observational period of > 365 days prior to the first prescription day of the study drugs and excluded patients with known prior exposure to any other statin (simvastatin, pravastatin, lovastatin, and fluvastatin), including crossover among the study drugs at any time before and within 180 days after the first prescription of the study drug. In this study, 2–4 mg of pitavastatin, 10–80 mg of atorvastatin, and 5–20 mg of rosuvastatin were used. We defined 10–20 mg of atorvastatin and 5–10 mg of rosuvastatin as moderate-intensity statin and 40–80 mg of atorvastatin and 20 mg of rosuvastatin as high-intensity statin according to the recent guidelines [23].

The index date was defined as the first day of study drug prescription. The target cohort was defined as patients who were first prescribed pitavastatin at any dose for > 180 consecutive days. The comparator cohort was defined as patients who were first prescribed atorvastatin or rosuvastatin (atorvastatin + rosuvastatin) at any dose for > 180 consecutive days. Continuous drug exposure was established by allowing gaps < 90 days between prescriptions. The cohort end date was defined as the date of the end of continuous study drug exposure, drug exposure of another statin during follow-up, or ascertainment of NODM. The time at risk of the study was defined from 180 days after the index date to 180 days after the cohort end date. Patients who met at least one of the following criteria at any days before and within 180 days after the index date were also excluded from both cohorts to confirm the absence of diabetes mellitus and impaired glucose tolerance: (1) a diagnosis of diabetes disorder including impaired glucose tolerance; (2) exposure to any oral hypoglycemic agent, glucagon-like pepetide-1 receptor agonists, or insulin; and (3) serum hemoglobin A1c (HbA1c) level ≥ 5.7%.

The primary outcome was the incidence of NODM 180 days after the index date. NODM was defined as the occurrence of at least one of the following criteria between 180 days after the index date and 180 days after the cohort end date: (1) diagnosis of diabetes mellitus as identified by the 10th version of the International Classification of Diseases (ICD); (2) prescription of any hypoglycemic agent, glucagon-like pepetide-1 receptor agonist, or insulin; and (3) serum HbA1c level ≥ 6.5%. The secondary outcomes included (1) the incidence of NODM in each group (any dose of pitavastatin, atorvastatin, and rosuvastatin), (2) the incidence of NODM with any dose of pitavastatin and high-intensity atorvastatin or rosuvastatin, and (3) the incidence of NODM with any dose of pitavastatin and moderate-intensity atorvastatin or rosuvastatin. In the analysis according to the statin intensities, we also excluded patients having dose changes between high and moderate-intensity statin during follow up.

### Statistical analysis

We performed our cohort study using the open-source OHDSI CohortMethod R package with large-scale analytics from the Cyclops R package [24, 25]. We used ATLAS version 2.7.5, and the analysis was performed using FEEDER-NET, a Korean health data platform based on the OMOP-CDM. We used large-scale propensity score matching to balance the target and comparator cohorts to reduce potential confounding due to an imbalance in baseline covariates. Covariates used in the propensity score model included age, sex, prior conditions, drugs observed during the 365 days and 30 days prior to study drug exposure, and Romano’s Adaptation of the Charlson comorbidity index [26]. Propensity scores were estimated using large-scale logistic regression models, and greedy search matching was used to match patients with a caliper of 0.2 times for the standard deviation of the propensity score distribution. We performed 1:2 propensity score matching (PSM) to compare pitavastatin with atorvastatin + rosuvastatin. Additionally, 1:1 PSM was used for comparing pitavastatin vs. atorvastatin, pitavastatin vs. rosuvastatin, atorvastatin vs. rosuvastatin, pitavastatin vs. moderate-intensity atorvastatin + rosuvastatin, pitavastatin vs. moderate-intensity atorvastatin, pitavastatin vs. moderate-intensity rosuvastatin, and pitavastatin vs. high-intensity atorvastatin + rosuvastatin. We conducted Cox regression analysis to examine the HR for NODM between the cohorts. Kaplan–Meier analysis was used to estimate the cumulative incidence of NODM. Incidence rates were determined per 1000 person-years by dividing the number of cases of NODM by the total number of person-years at risk. Two-sided *P*-values < 0.05 were considered statistically significant.

After conducting identical analytic process 10 databases with the single execute-to-end dedicated R package, we aggregated results of 10 databases by meta-analysis. The assessment for statistical tests of heterogeneity was calculated using the *χ*^2^ and *I*^2^ statistics. When there was no significant result for heterogeneity (P > 0.10, *I*^2^ < 50%), a fixed-effects model was used; otherwise, a random-effects model was used. However, we reported results from both, fixed- and random-effects models as a sensitivity analysis. All analyses were performed using R statistical software (version 3.6.1; R Foundation for Statistical Computing).

## Results

**Fig. 1:**
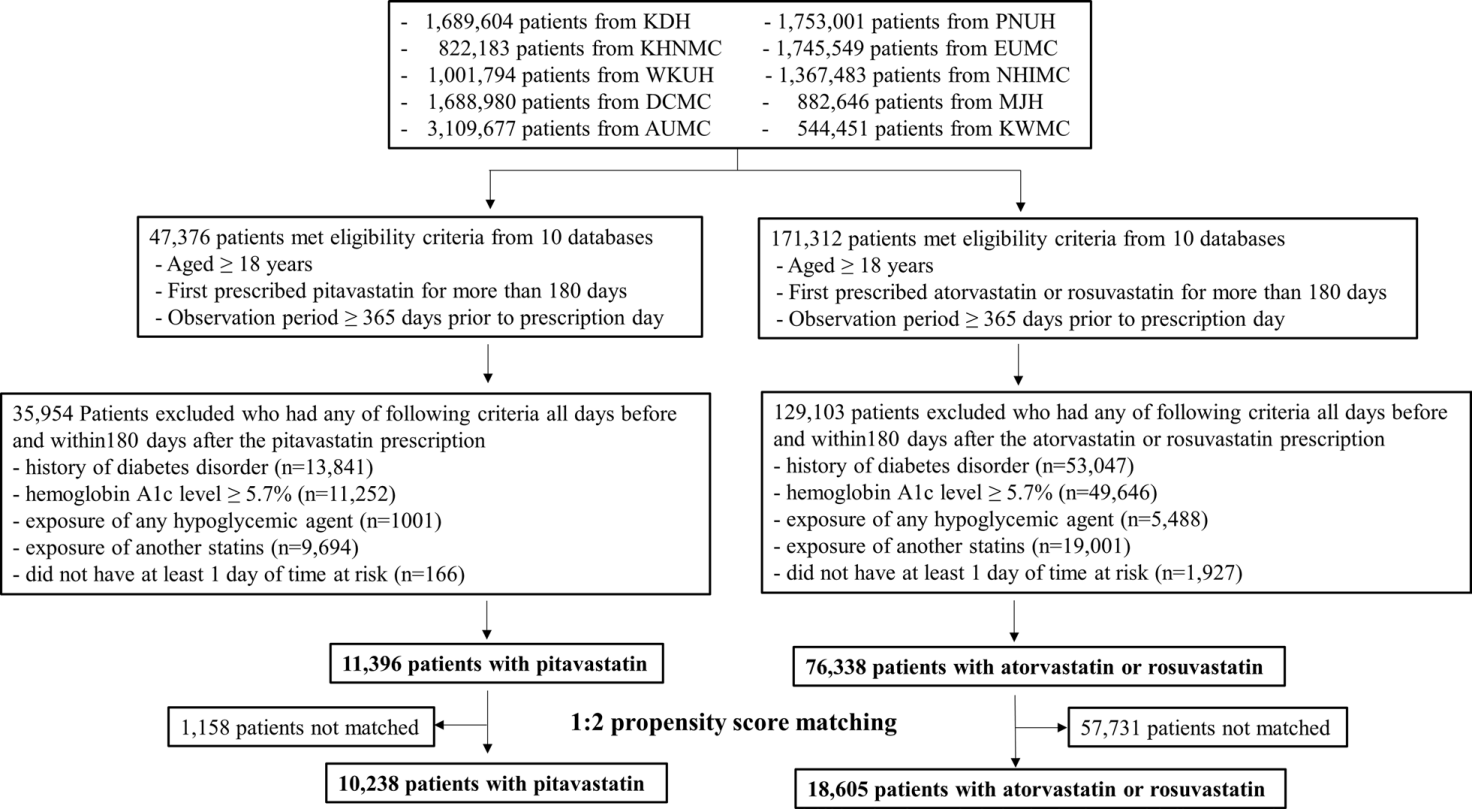
Study flowchart of patients using pitavastatin versus atorvastatin or rosuvastatin

**Fig. 2:**
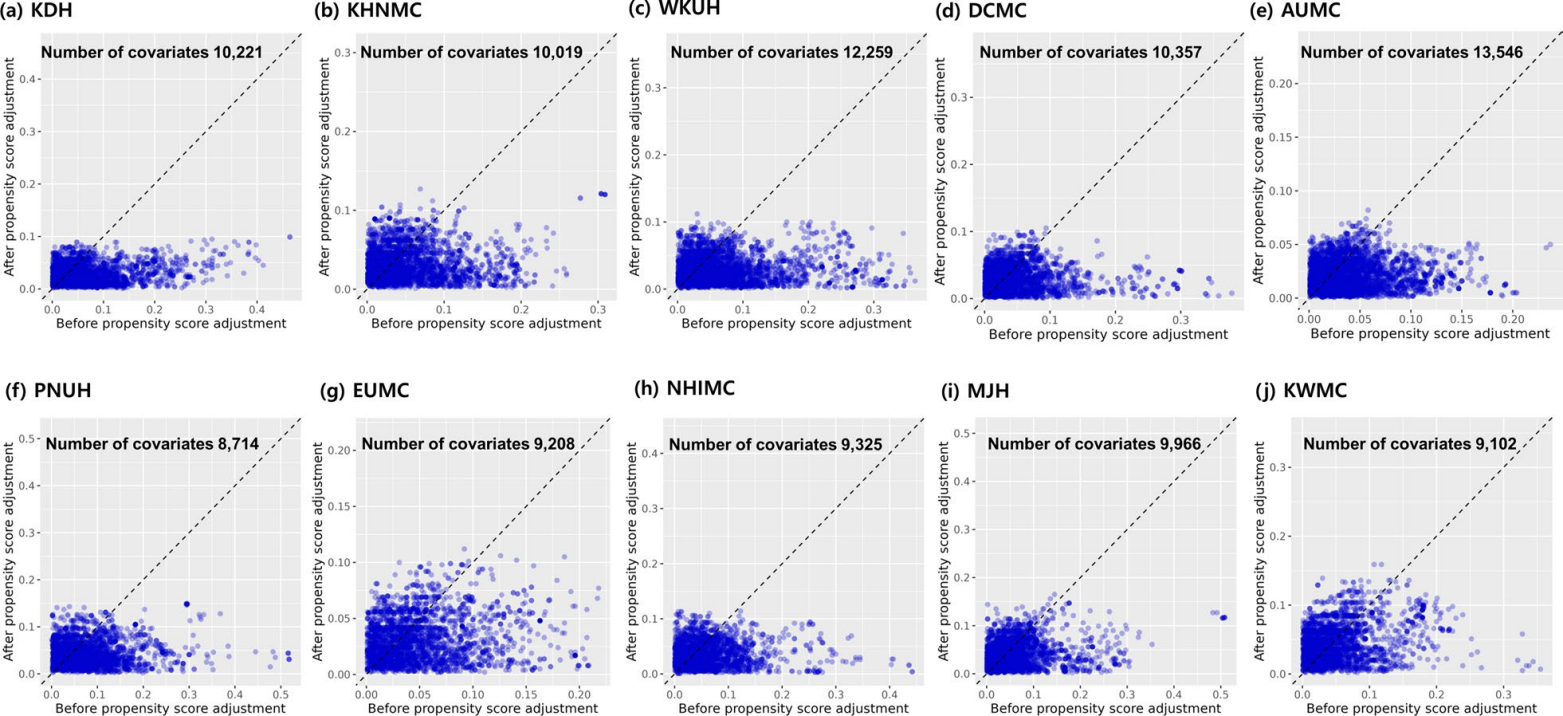
Covariate balance plot before and after propensity score matching across 10 databases

A total of 87,734 patients across the 10 databases were included in the present study (11,396 patients were new users of pitavastatin [17,944 person-years of follow-up] and 76,338 patients were new users of atorvastatin + rosuvastatin [137,966 person-years of follow-up]) (Fig. 1). We performed 1:2 PSM to compare the two groups. From 8714 (PNUH) to 13,546 (AUMC) baseline covariates were matched in 10 databases and most of the standardized mean differences were lesser than 0.1 after PSM (Fig. 2). In total, 10,238 patients treated with pitavastatin (15,998 person-years of follow-up) and 18,605 patients treated with atorvastatin + rosuvastatin (33,477 person-years of follow-up) were pooled after 1:2 PSM.

Table 1 shows the baseline characteristics of patients in the KDH cohort before and after PSM. The atorvastatin + rosuvastatin group had a higher Charlson comorbidity index score and incidence of ischemic heart disease and cerebrovascular disease before PSM, but the baseline covariables were well-balanced after PSM. Across all databases, similar results were obtained (Additional file 1: Table S1–S9).

**Table 1:** Baseline characteristics of patients with pitavastatin vs. atorvastatin or rosuvastatin in KDH cohort

	Before PS adjustment			After PS adjustment		
	Pitavastatin (n = 1652)	Atorvastatin + rosuvastatin (n = 5787)	Std. diff	Pitavastatin (n = 1154)	Atorvastatin + rosuvastatin (n = 1876)	Std. diff
Age group						
20–24	< 0.3	0.3	− 0.01	< 0.4	< 0.2	− 0.03
25–29	0.4	0.8	− 0.05	0.4	0.9	− 0.06
30–34	1.0	1.7	− 0.06	1.0	1.3	− 0.02
35–39	1.7	3.3	− 0.10	1.7	1.9	− 0.01
40–44	4.6	4.7	0.00	4.9	5.2	− 0.01
45–49	11.9	8.2	0.13	9.4	10.6	− 0.04
50–54	23.2	12.9	0.27	17.4	21.0	− 0.09
55–59	20.4	15.7	0.12	20.5	17.1	0.09
60–64	14.5	14.1	0.01	16.0	15.4	0.02
65–69	8.5	11.7	− 0.11	10.7	9.9	0.02
70–74	7.1	10.7	− 0.13	9.0	8.3	0.03
75–79	3.6	7.6	− 0.18	4.9	4.2	0.03
80–84	1.8	5.2	− 0.19	2.5	2.1	0.03
85–89	0.8	2.4	− 0.12	1.2	1.4	− 0.02
90–94	< 0.3	0.7	− 0.06	< 0.4	0.5	− 0.04
Female	76.3	54.8	0.46	67.3	71.9	− 0.10
Charlson comorbidity index^a^	0.52	0.71	− 0.19	0.56	0.52	0.03
Hyperlipidemia	17.5	17.3	0.00	17.1	17.5	− 0.01
Hypertensive disorder	26.7	29.0	− 0.05	27.1	26.0	0.02
Atrial fibrillation	1.0	1.6	− 0.05	1.4	1.1	0.03
Cerebrovascular disease	2.9	7.7	− 0.21	4.0	3.6	0.02
Heart failure	1.7	3.5	− 0.11	2.3	2.0	0.02
Ischemic heart disease	5.1	13.7	− 0.30	7.0	5.8	0.05
Chronic liver disease	< 0.3	0.2	0.00	< 0.4	< 0.2	0.02
Chronic obstructive lung disease	0.7	1.3	− 0.06	1.0	0.8	0.01
Renal impairment	1.2	2.1	− 0.07	1.6	1.3	0.02
Gastroesophageal reflux disease	6.2	5.8	0.01	6.0	5.8	0.01
Osteoarthritis	1.0	1.5	− 0.04	0.7	1.1	− 0.04
Dementia	1.0	1.5	− 0.04	0.7	1.1	− 0.04
Depressive disorder	0.5	2.3	− 0.15	0.8	0.9	− 0.01
Schizophrenia	1.7	2.3	− 0.04	1.7	1.8	− 0.01
Visual system disorder	0.4	0.1	0.06	0.4	< 0.2	0.08
Medication use						
RAS blocker	15.9	24.0	− 0.20	21.1	18.3	0.07
Beta blocker	17.9	23.8	− 0.14	22.0	21.3	0.02
Calcium channel blockers	10.9	16.0	− 0.15	13.5	11.9	0.05
Diuretics	15.3	21.6	− 0.16	17.6	16.0	0.04
Antibiotics use	26.0	24.3	0.04	17.3	18.6	− 0.03
NSAIDs use	29.6	28.3	0.03	22.0	23.6	− 0.04
Drugs for acid related disorders	30.8	42.7	− 0.25	29.9	28.1	0.04
Drugs for obstructive airway diseases	3.0	5.1	− 0.11	3.1	3.6	− 0.02
Immunosuppressants	1.3	1.7	− 0.04	1.5	0.8	0.06

PS, propensity score; RAS, renin-angiotensin system; NSAIDs, nonsteroidal anti-inflammatory drugs

^a^Romano’s Adaptation of the Charlson Comorbidity Index was used and presented as the mean value. All other variables are presented as a percentage of the sample size

**Fig. 3:**
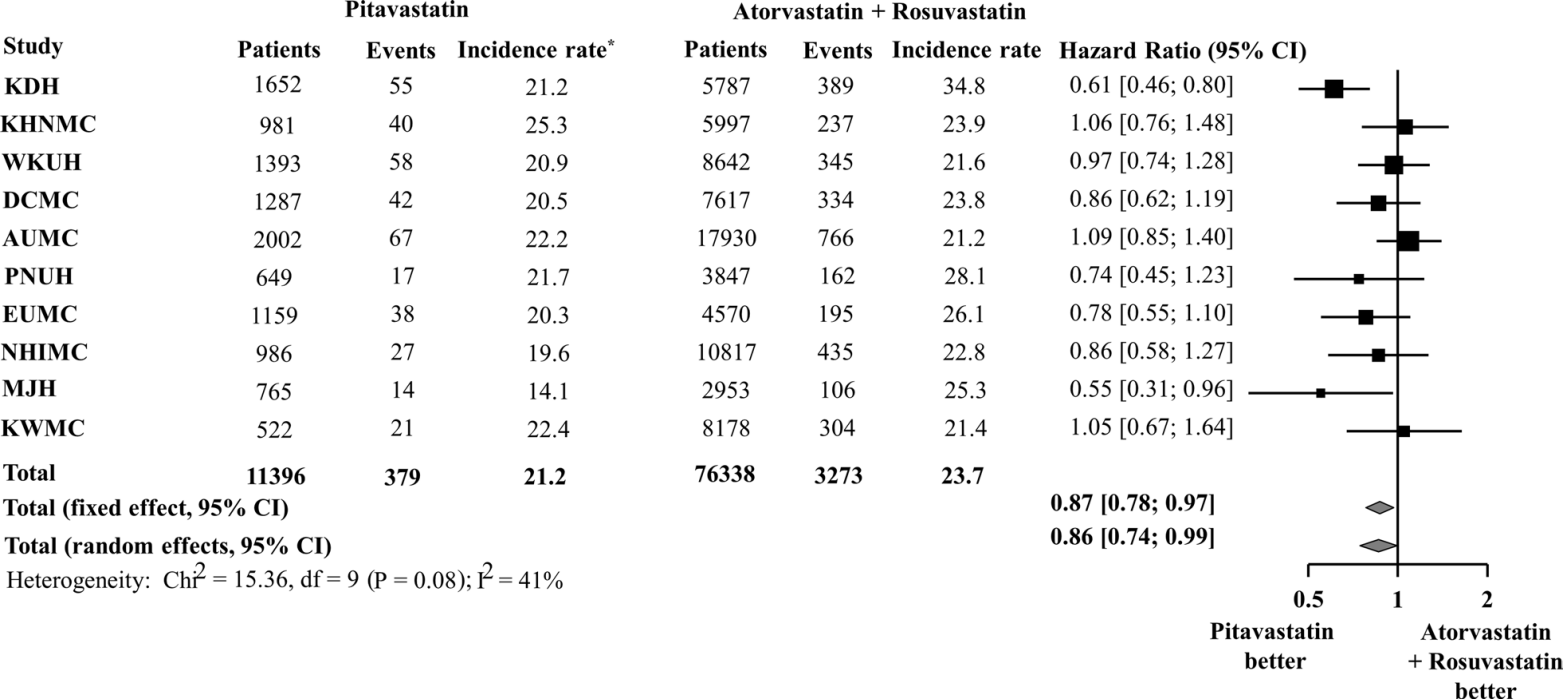
Risk of new-onset diabetes mellitus between pitavastatin vs. atorvastatin or rosuvastatin in the overall population. *Incidence rate per 1000 person-years

**Fig. 4:**
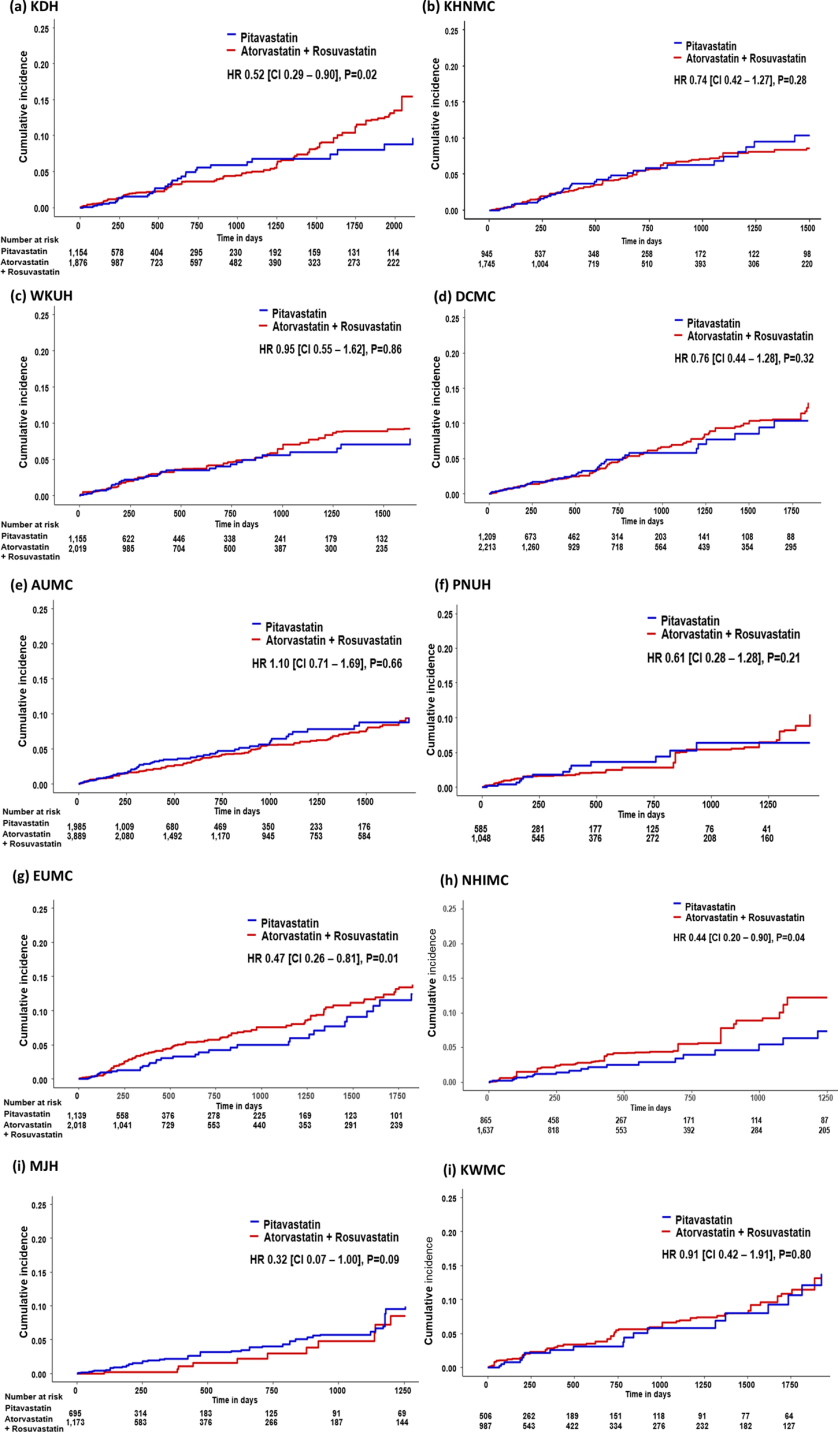
Kaplan–Meier plots comparing risk of new-onset diabetes mellitus from pitavastatin vs. atorvastatin or rosuvastatin across 10 databases after propensity score matching

**Fig. 5:**
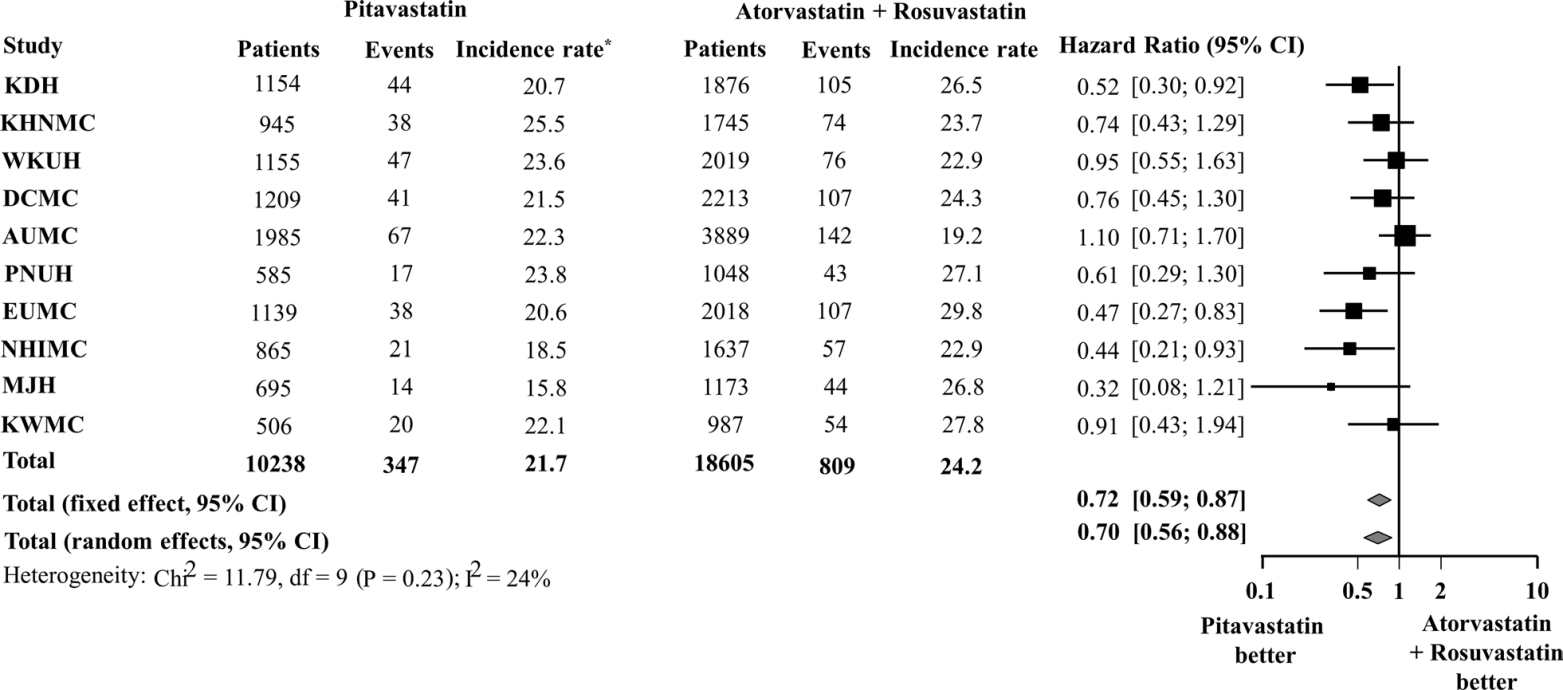
Risk of new-onset diabetes mellitus between pitavastatin vs. atorvastatin or rosuvastatin after propensity score matching. *Incidence rate per 1000 person-years

Figure 3 shows the results comparing the risk of NODM between those taking pitavastatin and those taking atorvastatin + rosuvastatin before PSM across the 10 databases. The HR (95% CI) for the meta-analytic estimate of the risk of NODM showed that pitavastatin was associated with a lower risk of NODM than atorvastatin + rosuvastatin (HR 0.86; 95% CI 0.74–0.99). After PSM, results from three databases (KDH, EUMC, and NHIMC) showed that the pitavastatin group had a significantly lower cumulative incidence of NODM than the atorvastatin + rosuvastatin group (Fig. 4). After pooling results from 10 databases, the incidence rate of NODM during pitavastatin use was 21.7 per 1000 person-years and that of atorvastatin + rosuvastatin use was 24.2 per 1000 person-years. The meta-analysis of HR from 10 databases demonstrated that the pitavastatin group had a statistically significant lower risk of NODM than the atorvastatin + rosuvastatin group (HR 0.72; 95% CI 0.59–0.87) (Fig. 5).

**Fig. 6:**
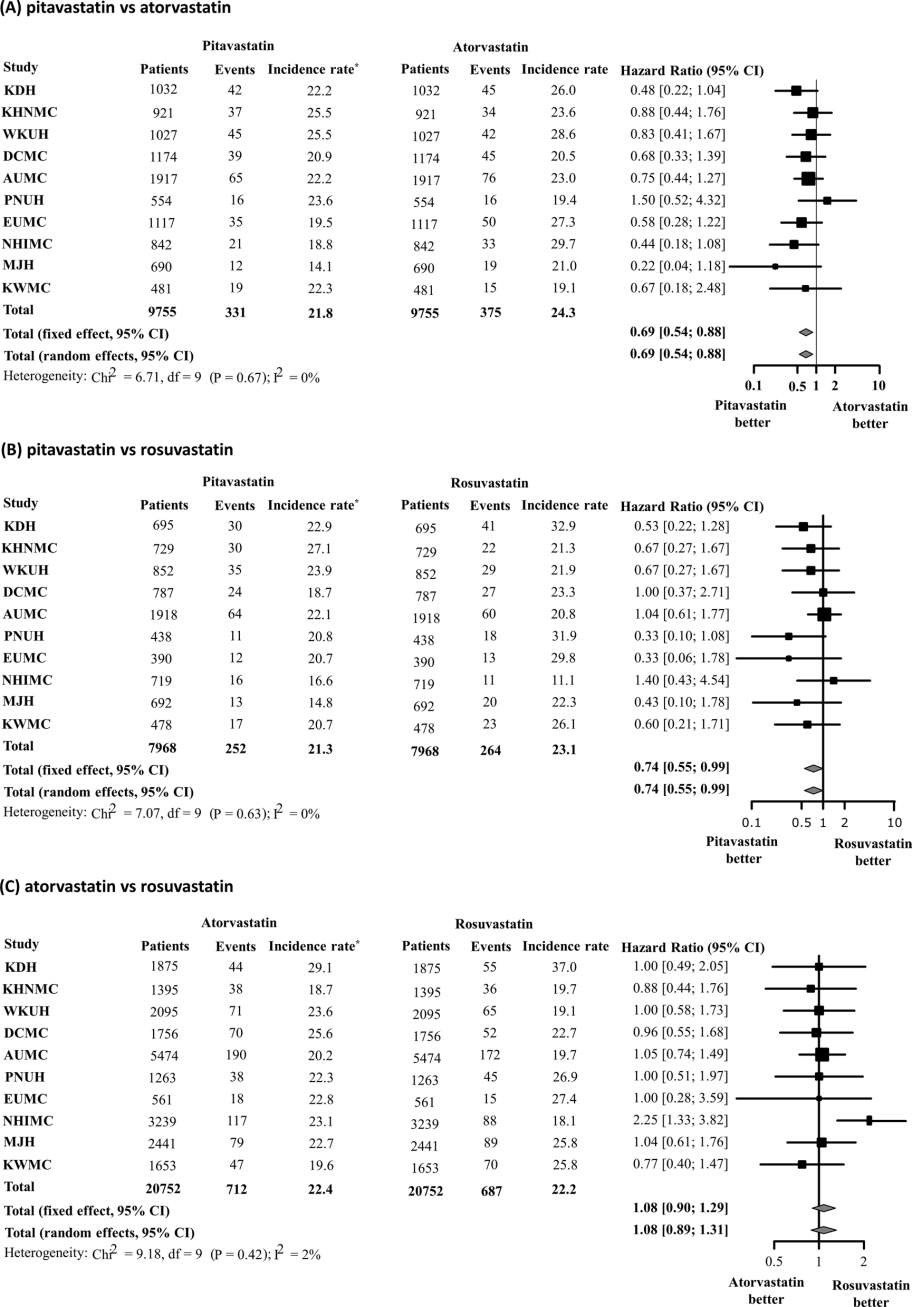
Risk of new-onset diabetes mellitus after 1:1 propensity score matching. **A** Pitavastatin vs. atorvastatin, **B** pitavastatin vs. rosuvastatin, **C** atorvastatin vs. rosuvastatin. *Incidence rate per 1000 person-years

**Fig. 7:**
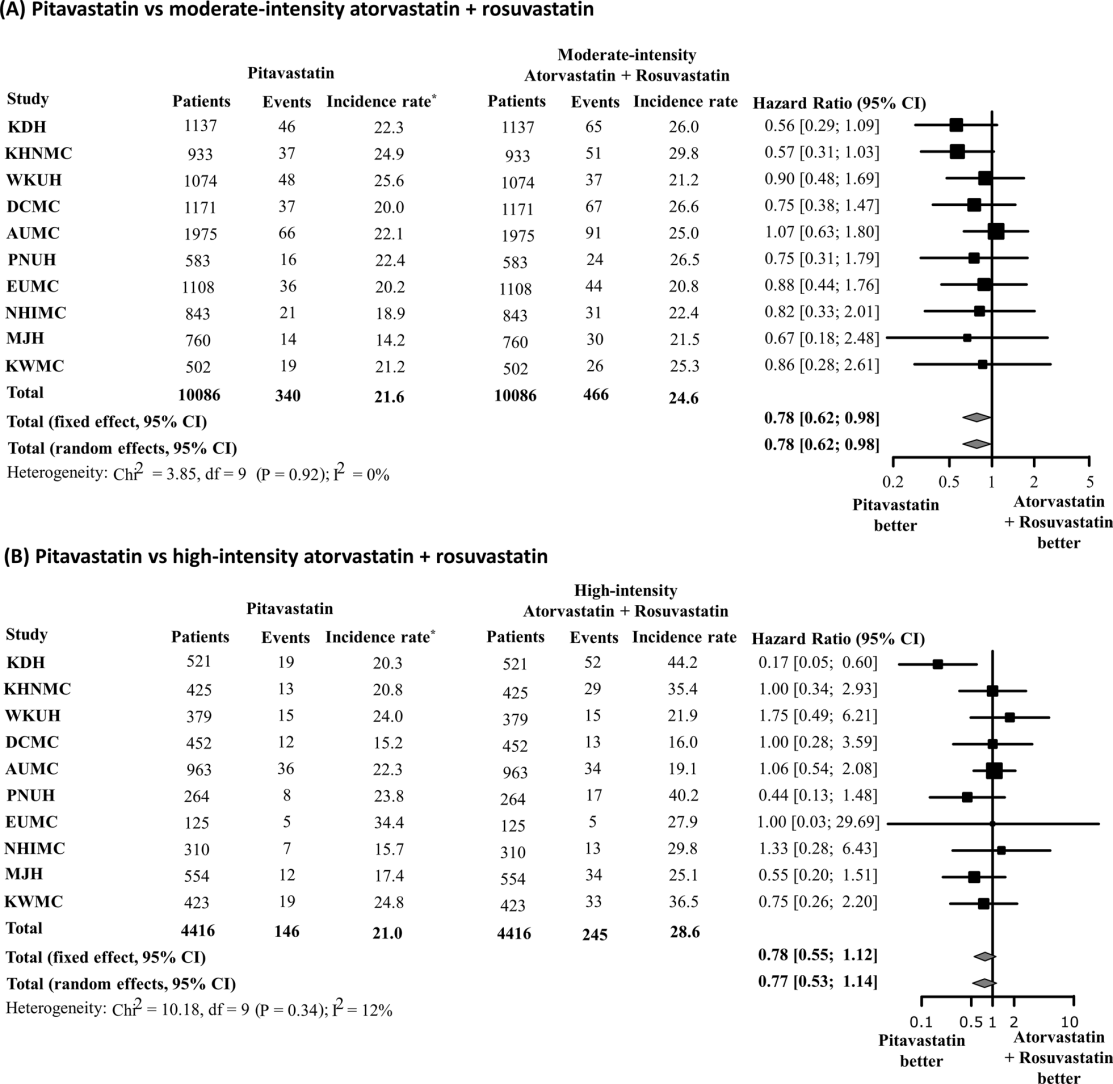
Risk of new-onset diabetes mellitus according to intensity of statin treatment. **A** All doses of pitavastatin vs. moderate-intensity atorvastatin or rosuvastatin, **B** all doses of pitavastatin vs. high-intensity atorvastatin or rosuvastatin. *Incidence rate per 1000 person-years

**Fig. 8:**
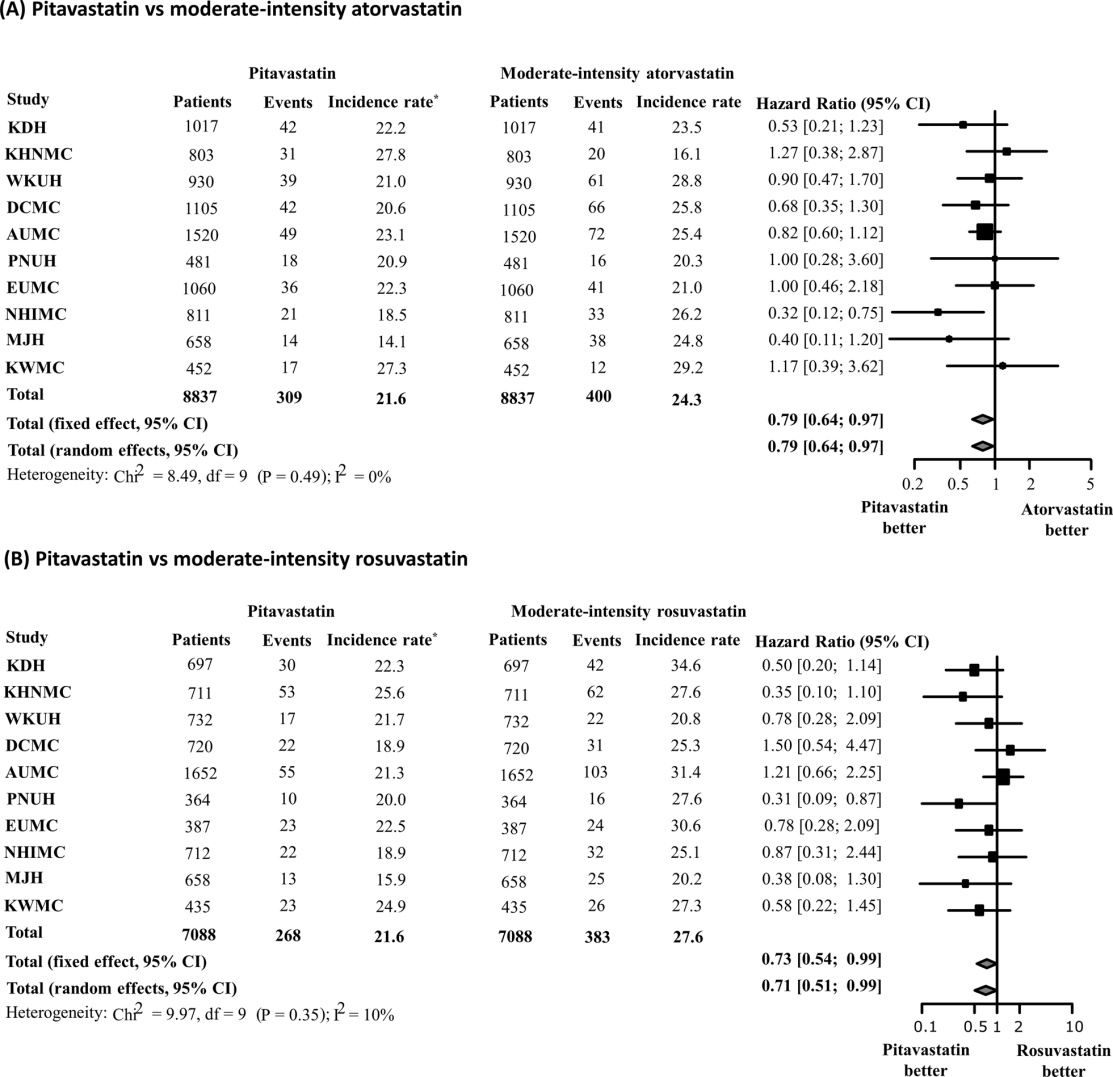
Comparison of risk of new-onset diabetes mellitus among moderate-intensity statins. **A** all doses of pitavastatin vs. moderate-intensity atorvastatin, **B** all doses of pitavastatin vs. moderate-intensity rosuvastatin. *Incidence rate per 1000 person-years

In the sub-analysis, we created five 1:1 propensity-score-matched cohorts to compare pitavastatin vs. atorvastatin, pitavastatin vs. rosuvastatin, atorvastatin vs. rosuvastatin, pitavastatin vs. moderate-intensity atorvastatin + rosuvastatin, pitavastatin vs. moderate-intensity atorvastatin, pitavastatin vs. moderate-intensity rosuvastatin, and pitavastatin vs. high-intensity atorvastatin + rosuvastatin in the same manner. The meta-analysis of HR showed that pitavastatin was associated with a lower risk of NODM than atorvastatin or rosuvastatin (pitavastatin vs. atorvastatin: HR 0.69; 95% CI 0.54–0.88; pitavastatin vs. rosuvastatin: HR 0.74; 95% CI 0.55–0.99) (Fig. 6). However, no significant difference was observed in the risk of NODM between the atorvastatin and rosuvastatin groups (HR 1.08; 95% CI 0.90–1.29). In addition, pitavastatin was associated with a lower risk of NODM compared to moderate-intensity atorvastatin + rosuvastatin (HR 0.78; 0.62–0.98); however, no significant difference was observed between pitavastatin and high-intensity atorvastatin + rosuvastatin (HR 0.78; 95% CI 0.55–1.12) (Fig. 7). A consistently low risk of NODM in pitavastatin users were observed when compared with each of moderate-intensity atorvastatin or rosuvastatin users (pitavastatin vs. moderate-intensity atorvastatin: HR 0.79; 95% CI 0.64–0.97; pitavastatin vs. moderate-intensity rosuvastatin: HR 0.73; 95% CI 0.54–0.99) (Fig. 8).

## Discussion

This is the first distributed network research focusing on NODM risk related to statins by head-to-head comparison using real-world clinical CDM data from 10 institutions. The present study showed that pitavastatin reduced the HR of NODM by 28% compared with atorvastatin + rosuvastatin. In sub-analysis, pitavastatin also had a significantly lower risk of NODM than each of atorvastatin or rosuvastatin. This effect was prominent in the comparison between pitavastatin and moderate-intensity atorvastatin or rosuvastatin, but there was no statistically significant difference in the comparison between pitavastatin and high-intensity atorvastatin or rosuvastatin. It is generally accepted that the benefits of statins for preventing cardiovascular disease far outweigh the risk of NODM; nevertheless, administration of statins with a lower risk of NODM is ideal for those at high-risk of diabetes [4, 27].

Several clinical trials and meta-analyses have revealed the effect of statins on NODM [3, 4]. The mechanism by which statins affect glucose homeostasis is not fully understood; however, several preclinical studies have demonstrated that statins may influence insulin signaling in peripheral tissues, resistance, and secretion, as well as pancreatic beta-cell function, and glucose metabolism [28, 29]. In contrast, there have been several experimental studies supplying evidence of a lower risk of NODM with pitavastatin. In an in vitro study, the reduction in the rate of insulin secretion in pancreatic islet β-cells treated with pitavastatin was less than that of those treated with atorvastatin or rosuvastatin, and cell viability was also better than that with other statins [28, 30]. Similarly, the reduction in coenzyme Q10 levels caused by statin treatment affects insulin secretion and abnormal glucose metabolism, but there is evidence that pitavastatin has minimal effects on coenzyme Q10 through its unique pharmacological mechanism [28, 31]. Moreover, the glucose uptake rate of human skeletal muscle after treatment with pitavastatin was better than that with atorvastatin or rosuvastatin [30]. In addition, adiponectin concentration is inversely correlated with insulin resistance and visceral obesity [32]. It has been reported that pitavastatin significantly increases adiponectin levels, while atorvastatin has neural effects on adiponectin levels and rosuvastatin has harmful effects. These findings could explain the lower risk of NODM after pitavastatin treatment [33, 34].

Clinical studies have suggested that pitavastatin may be associated with a lower risk of NODM than other statins. A network meta-analysis of 29 randomized clinical trials by Thakker et al. reported that pitavastatin had the lowest risk of NODM among six statins (rosuvastatin, atorvastatin, pravastatin, simvastatin, lovastatin, and pitavastatin) compared to the placebo; however, there was no significant difference between pitavastatin and atorvastatin or rosuvastatin [16]. Moreover, in a real-world cohort study of Asian patients, pitavastatin tended to have a lower HR for NODM than other statins, though the difference was not statistically significant [7]. A recently reported single-center study also showed that atorvastatin and rosuvastatin had a significantly higher risk of NODM than pitavastatin [35]. Nevertheless, there is a counterargument for these results. Cho et al. performed a single-center, retrospective study of 3680 patients and reported that pitavastatin had the highest HR for NODM than other statins with simvastatin as a reference [17]. As such, previous results from clinical studies are controversial, and data for the direct comparison of the risk of diabetes between pitavastatin and other statins is limited.

The present study is a meta-analysis of the results from CDM-converted multicenter EMR data analysis performed in large-scale PS matched cohorts. It has the advantage of reducing the risk of confounding bias and providing high-level evidence for the direct comparison of the risk of diabetes among the study drugs. Moreover, the present study was conducted on patients who had an observation period of ≥ 1 year before statin administration with no previous exposure to any statins. The active-comparator and new-user designs implemented in the present study could mitigate the methodological limitations of observational studies, such as immortal-time bias [36]. Moreover, to remove protopathic bias, NODM was defined as patients diagnosed at least 180 days (lag period) after the index date. Another strength of the present study was that it excluded patients who had serum HbA1c levels ≥ 5.7% at any point before and within 180 days after enrollment, which increased the reliability of the causal relationship between statins and NODM. In addition, we performed common statistical analysis on the databases using the same analytic R code, and the results of the study had relatively good reproducibility across all 10 databases. Future research could be expanded to large datasets.

Pitavastatin was classified as a moderate-intensity statin, and Choi et al. reported that it is associated with a lower incidence of NODM in patients with acute myocardial infarction compared to moderate-intensity atorvastatin or rosuvastatin [37]. The present study also showed that pitavastatin had a lower risk of NODM than moderate-intensity atorvastatin or rosuvastatin. However, the difference in NODM risk between pitavastatin and high-intensity atorvastatin or rosuvastatin did not reach statistical significance in the present study. The numerically highest difference in the incidence rate of NODM was observed between the pitavastatin and high-intensity atorvastatin or rosuvastatin groups (incidence rate of 21.0 vs. 28.6 per 1000 person-years, respectively). In general, high-intensity statins are associated with a greater risk of NODM than low-to-moderate-intensity statins [4]. Considering the wide range of confidence intervals, a possible explanation for our null finding is the relatively small sample size related to statistical power. Because high-intensity statins were often used following moderate-intensity statin therapy in routine clinical practice, there were a few patients who were new-user of high-intensity atorvastatin or rosuvastatin. In addition, the indications vary according to statin intensity, there was a disparity in the distribution of propensity scores between the pitavastatin and high-intensity atorvastatin or rosuvastatin groups. In this study, only 4416 patients were assigned to each group after 1:1 PSM, which was deemed the main reason for the lack of statistical significance between pitavastatin and high-intensity atorvastatin or rosuvastatin.

Since comparison of active treatment with placebo inevitably induces substantial bias in observational setting, we did not evaluate the risk of NODM in pitavastatin uses compared to non-statin users [36]. Some studies have shown that pitavastatin did not adversely affect HbA1c level [13, 38]. Recent retrospective study using the Korean insurance claims database reported that the risk of NODM was the largest for atorvastatin followed by rosuvastatin among individual statins when compared to non-statin users, but pitavastatin was not associated with increasing risk of NODM [39]. However, only 27 patients prescribed with pitavastatin were enrolled in that study. Another retrospective study showed that pitavastatin had a lower HR for NODM than atorvastatin or rosuvastatin, but which was significantly higher than that of the non-statin user group [40]. Therefore, further research is needed to investigate the risk of NODM in pitavastatin users compared to non-statin users.

Our study has several limitations. First, owing to the observational nature of the study design, residual confounding factors may have influenced the study results despite adjustment with large-scale propensity score matching. Moreover, some information regarding patients receiving antidiabetic drugs or statins at hospitals other than those included in the study may have been missed. Second, the definition of NODM differs from recent guidelines [41]. Since we could not confirm fasting blood glucose or two-hour plasma glucose during an oral glucose tolerance test, we used ICD-10 code, HbA1c, and diabetic drug history as criteria for NODM. Third, this study used an aggregate meta-analysis approach without pooling individual data. While our combined data sources provided the population size needed to demonstrate a statistically significant effect in favor of pitavastatin, only three databases had significant results separately. However, a previous study showed that aggregate meta-analysis yields estimate that are at least as precise and accurate as the pooled individual dataset [42].

## Conclusions

In conclusion, pitavastatin was associated with a lower risk of NODM than atorvastatin or rosuvastatin in patients who were newly treated with statins. This study could guide the selection of statins for patients with a high risk of diabetes in clinical practice.

## Supplementary Information

**Additional file 1**: **Table S1.** Baseline characteristics of patients with pitavastatin vs. atorvastatin or rosuvastatin in KHNMC cohort. **Table S2.** Baseline characteristics of patients with pitavastatin vs. atorvastatin or rosuvastatin in WKUH cohort. **Table S3.** Baseline characteristics of patients with pitavastatin vs. atorvastatin or rosuvastatin in DCMC cohort. **Table S4.** Baseline characteristics of patients with pitavastatin vs. atorvastatin or rosuvastatin in AUMC cohort. **Table S5.** Baseline characteristics of patients with pitavastatin vs. atorvastatin or rosuvastatin in PNUH cohort. **Table S6.** Baseline characteristics of patients with pitavastatin vs. atorvastatin or rosuvastatin in EUMC cohort. **Table S7.** Baseline characteristics of patients with pitavastatin vs. atorvastatin or rosuvastatin in NHIMC cohort. **Table S8.** Baseline characteristics of patients with pitavastatin vs. atorvastatin or rosuvastatin in MJH cohort. **Table S9.** Baseline characteristics of patients with pitavastatin vs. atorvastatin or rosuvastatin in KWMC cohort.

## Data Availability

The datasets generated and/or analyzed during the current study are available from the corresponding author upon reasonable request.

## Abbreviations

NODM: New-onset diabetes mellitus
HR: Hazard ratio
CDM: Common data model
HbA1c: Hemoglobin A1c
ICD: International classification of disease
PSM: Propensity score matching
