# Supplementary material for: Impact of pitavastatin on new-onset diabetes mellitus compared to atorvastatin and rosuvastatin: a distributed network analysis of 10 real-world databases
Source: Cardiovasc Diabetol. 2022 May 23;21:82. doi: 10.1186/s12933-022-01524-6 (PMC9128291; doi:10.1186/s12933-022-01524-6)
Supplement: Supplementary file 1 — Additional file 1: Table S1. Baseline characteristics of patients with pitavastatin vs. atorvastatin or rosuvastatin in KHNMC cohort. Table S2. Baseline characteristics of patients with pitavastatin vs. atorvastatin or rosuvastatin in WKUH cohort. Table S3. Baseline characteristics of patients with pitavastatin vs. atorvastatin or rosuvastatin in DCMC cohort. Table S4. Baseline characteristics of patients with pitavastatin vs. atorvastatin or rosuvastatin in AUMC cohort. Table S5. Baseline characteristics of patients with pitavastatin vs. atorvastatin or rosuvastatin in PNUH cohort. Table S6. Baseline characteristics of patients with pitavastatin vs. atorvastatin or rosuvastatin in EUMC cohort. Table S7. Baseline characteristics of patients with pitavastatin vs. atorvastatin or rosuvastatin in NHIMC cohort. Table S8. Baseline characteristics of patients with pitavastatin vs. atorvastatin or rosuvastatin in MJH cohort. Table S9. Baseline characteristics of patients with pitavastatin vs. atorvastatin or rosuvastatin in KWMC cohort. [file 12933_2022_1524_MOESM1_ESM.pdf]

Table S1. Baseline characteristics of patients with pitavastatin vs. atorvastatin or rosuvastatin in KHNMC cohort.

	Before PS adjustment			After PS adjustment		
	Pitavastatin (n=981)	Atorvastatin+ Rosuvastatin (n=5997)	Std. diff	Pitavastatin (n=945)	Atorvastatin+ Rosuvastatin (n=1745)	Std. diff
Age group						
20-24	<0.5	0.3	-0.04	<0.5	0.4	-0.06
25-29	<0.5	0.8	-0.05	<0.5	1.1	-0.07
30-34	1.2	1.4	-0.02	1.2	1.5	-0.03
35-39	4.3	3.2	0.06	4.1	4.4	-0.01
40-44	5.6	5.1	0.02	5.5	5.1	0.02
45-49	8.8	9.1	-0.01	8.9	9.9	-0.04
50-54	16.1	13.3	0.08	16.2	15.8	0.01
55-59	19.3	17.2	0.05	19.2	17.2	0.05
60-64	16.3	14.7	0.04	16.6	15.9	0.02
65-69	10.5	11.2	-0.02	10.4	11.3	-0.03
70-74	6.7	9.7	-0.11	6.7	7.1	-0.02
75-79	4.8	6.8	-0.08	4.9	5.0	0.00
80-84	4.0	4.5	-0.02	4.0	3.4	0.03
85-89	1.5	2.1	-0.04	1.6	1.4	0.01
90-94	<0.5	0.5	-0.06	<0.5	0.3	-0.02
Female	65.1	56.3	0.18	64.1	66.6	-0.05
Charlson comorbidity index*	0.38	0.58	-0.20	0.38	0.43	-0.04
Hyperlipidemia	43.2	37.0	0.13	42.4	45.9	-0.07
Hypertensive disorder	26.7	29.0	-0.05	27.1	26.0	0.02
Atrial fibrillation	0.9	1.8	-0.07	1.0	1.3	-0.03
Cerebrovascular disease	5.2	7.9	-0.11	5.4	4.0	0.07
Heart disease	15.4	24.7	-0.23	16.0	14.1	0.05
Heart failure	15.4	24.7	-0.23	16.0	14.1	0.05
Ischemic heart disease	6.2	12.4	-0.21	6.6	5.4	0.05

Chronic liver disease	<0.5	0.1	0.05	<0.5	<0.3	0.06
Chronic obstructive lung disease	1.4	1.2	0.02	1.5	0.7	0.07
Renal impairment	1.1	2.8	-0.12	1.2	1.1	0.00
Gastroesophageal reflux disease	4.5	4.1	0.02	4.6	4.5	0.00
Osteoarthritis	1.8	1.7	0.01	1.9	1.5	0.03
Dementia	1.9	1.3	0.05	2.0	1.2	0.06
Depressive disorder	1.3	1.9	-0.05	1.3	1.9	-0.05
Visual system disorder	5.1	5.5	-0.01	5.3	5.1	0.01
Medication use						
RAS blocker	16.7	22.9	-0.15	16.9	15.3	0.04
Beta blocker	15.2	19.8	-0.12	14.9	14.6	0.01
Calcium channel blockers	11.4	18.4	-0.20	10.9	8.5	0.08
Diuretics	11.3	13.5	-0.07	11.1	10.0	0.04
Antibiotics	19.0	23.3	-0.11	18.7	16.8	0.05
NSAIDs	23.2	0.09	25.8	29.1	-0.07	23.2
Drugs for acid related disorders	55.7	58.0	-0.05	55.2	54.9	0.01
Drugs for obstructive airway diseases	5.7	5.9	-0.01	5.8	4.8	0.05
Immunosuppressants	3.7	1.5	0.14	3.5	3.8	-0.02

*Romano's Adaptation of the Charlson comorbidity index was used and presented as mean value. All other variables are presented as percent of the sample size.

PS, propensity score; RAS, renin-angiotensin system; NSAIDs, non-steroidal anti-inflammatory drugs

Table S2. Baseline characteristics of patients with pitavastatin vs. atorvastatin or rosuvastatin in WKUH cohort.

	Before PS adjustment			After PS adjustment		
	Pitavastatin (n=1393)	Atorvastatin+ Rosuvastatin (n=8642)	Std. diff	Pitavastatin (n=1155)	Atorvastatin+ Rosuvastatin (n=2019)	Std. diff
Age group						
20-24	0.5	0.2	0.05	0.4	0.5	-0.01
25-29	1.0	0.5	0.06	0.9	0.8	0.00
30-34	2.1	0.9	0.10	2.2	1.8	0.03
35-39	3.2	2.1	0.07	3.0	2.9	0.01
40-44	5.4	4.6	0.04	5.3	6.3	-0.04
45-49	8.6	7.5	0.04	7.9	8.5	-0.02
50-54	13.4	11.8	0.05	13.9	13.7	0.00
55-59	16.5	14.2	0.06	16.2	15.9	0.01
60-64	12.9	13.4	-0.02	12.6	12.3	0.01
65-69	11.6	13.3	-0.05	11.9	12.1	-0.01
70-74	10.7	12.3	-0.05	10.7	10.2	0.02
75-79	8.1	10.6	-0.09	8.7	8.4	0.01
80-84	4.0	5.8	-0.08	4.2	4.5	-0.01
85-89	1.6	2.3	-0.06	1.7	1.6	0.01
90-94	<0.4	0.4	-0.04	<0.4	0.3	-0.01
Female	52.8	49.6	0.06	51.7	52.1	-0.01
Charlson comorbidity index*	1.02	0.91	0.08	0.89	0.94	-0.03
Hyperlipidemia	32.2	31.4	0.02	32.3	32.5	0.00
Hypertensive disorder	39.0	43.5	-0.09	40.0	39.7	0.01
Atrial fibrillation	5.4	2.9	0.12	5.7	6.0	-0.01
Cerebrovascular disease	5.5	11.3	-0.21	6.3	4.9	0.06
Peripheral vascular disease	3.9	0.9	0.20	1.6	1.9	-0.02
Heart failure	7.6	3.1	0.20	5.7	7.3	-0.06

Ischemic heart disease	7.7	19.7	-0.35	9.1	8.2	0.03
Chronic liver disease	0.7	0.5	0.03	0.8	0.3	0.06
Chronic obstructive lung disease	1.1	1.4	-0.02	1.0	1.4	-0.04
Renal impairment	7.6	2.1	0.26	6.5	8.3	-0.07
Gastroesophageal reflux disease	3.9	2.3	0.09	3.5	3.4	0.01
Osteoarthritis	2.1	1.8	0.03	2.0	1.8	0.01
Dementia	1.3	3.3	-0.14	1.3	1.7	-0.03
Depressive disorder	2.8	2.9	-0.01	2.3	2.6	-0.02
Visual system disorder	5.1	5.2	-0.01	4.8	4.9	0.00
Medication use						
RAS blocker	29.1	22.5	0.15	27.2	29.1	-0.04
Beta blocker	24.7	24.8	0.00	25.1	25.8	-0.02
Calcium channel blockers	20.1	24.7	-0.11	20.6	19.7	0.02
Diuretics	22.8	19.2	0.09	17.8	19.2	-0.04
Antibiotics use	24.3	26.1	-0.04	22.0	22.9	-0.02
NSAIDs use	23.0	22.7	0.01	18.7	20.6	-0.05
Drugs for acid related disorders	51.4	59.9	-0.17	49.7	49.0	0.01
Drugs for obstructive airway diseases	5.7	6.0	-0.01	5.5	5.2	0.01
Immunosuppressants	5.2	0.8	0.26	2.0	3.2	-0.08

*Romano's Adaptation of the Charlson comorbidity index was used and presented as mean value. All other variables are presented as percent of the sample size.

PS, propensity score; RAS, renin-angiotensin system; NSAIDs, non-steroidal anti-inflammatory drugs

Table S3. Baseline characteristics of patients with pitavastatin vs. atorvastatin or rosuvastatin in DCMC cohort.

	Before PS adjustment			After PS adjustment		
	Pitavastatin (n=1287)	Atorvastatin+ Rosuvastatin (n=7617)	Std. diff	Pitavastatin (n=1209)	Atorvastatin+ Rosuvastatin (n=2213)	Std. diff
Age group						
20-24	0.4	0.2	0.03	0.4	<0.2	0.05
25-29	<0.4	0.4	-0.02	<0.4	0.3	-0.01
30-34	0.7	0.8	-0.01	0.8	1.0	-0.02
35-39	2.3	1.8	0.04	2.1	2.0	0.01
40-44	4.2	3.3	0.05	4.0	3.6	0.02
45-49	7.3	6.7	0.03	7.3	6.7	0.02
50-54	11.5	12.5	-0.03	11.7	12.2	-0.02
55-59	15.0	15.5	-0.01	15.1	16.1	-0.03
60-64	15.0	15.0	0.00	15.5	14.9	0.01
65-69	16.4	13.4	0.08	16.4	17.6	-0.03
70-74	11.0	12.8	-0.05	10.9	10.7	0.01
75-79	9.1	10.3	-0.04	9.1	8.8	0.01
80-84	4.7	5.2	-0.03	4.5	3.8	0.04
85-89	1.5	1.7	-0.02	1.6	2.0	-0.03
90-94	<0.4	0.3	-0.02	<0.4	<0.2	0.04
Female	64.3	57.4	0.14	64.4	66.0	-0.04
Charlson comorbidity index*	1.05	1.00	0.04	1.03	1.05	-0.01
Hyperlipidemia	68.3	63.0	0.11	70.4	70.6	0.00
Hypertensive disorder	37.5	32.1	0.11	36.9	38.7	-0.04
Atrial fibrillation	6.3	3.9	0.11	5.6	6.9	-0.05
Cerebrovascular disease	12.5	13.3	-0.02	12.7	13.7	-0.03
Peripheral vascular disease	16.5	12.0	0.13	15.3	17.3	-0.05
Heart failure	8.3	7.7	0.02	8.0	7.8	0.01

Ischemic heart disease	14.7	23.8	-0.23	14.5	12.9	0.05
Chronic liver disease	0.6	0.5	0.02	0.6	0.3	0.04
Chronic obstructive lung disease	1.9	1.8	0.01	1.7	1.6	0.00
Renal impairment	1.3	1.5	-0.01	1.3	1.6	-0.02
Gastroesophageal reflux disease	9.0	8.4	0.02	8.0	8.6	-0.02
Osteoarthritis	5.6	2.9	0.14	4.4	5.0	-0.03
Dementia	2.1	2.6	-0.03	2.1	2.2	-0.01
Depressive disorder	3.3	3.9	-0.03	3.2	4.1	-0.04
Visual system disorder	7.3	6.2	0.05	7.6	7.4	0.01
Medication use						
RAS blocker	16.6	11.7	0.14	15.5	16.1	-0.02
Beta blocker	30.0	32.2	-0.05	27.4	26.8	0.01
Calcium channel blockers	16.1	17.8	-0.05	16.1	16.2	0.00
Antibiotics use	13.6	14.9	-0.04	13.4	12.8	0.02
NSAIDs	28.2	24.7	0.08	28.1	29.2	-0.02
Drugs for acid related disorders	32.2	36.8	-0.10	31.3	31.9	-0.01
Drugs for obstructive airway diseases	5.9	4.3	0.07	5.5	5.3	0.01
Immunosuppressants	5.6	3.3	0.12	5.9	6.1	-0.01

*Romano's Adaptation of the Charlson comorbidity index was used and presented as mean value. All other variables are presented as percent of the sample size.

PS, propensity score; RAS, renin-angiotensin system; NSAIDs, non-steroidal anti-inflammatory drugs

Table S4. Baseline characteristics of patients with pitavastatin vs. atorvastatin or rosuvastatin in AUMC cohort.

	Before PS adjustment			After PS adjustment		
	Pitavastatin (n=2002)	Atorvastatin + Rosuvastatin (n=17930)	Std. diff	Pitavastatin (n=1985)	Atorvastatin + Rosuvastatin (n=3889)	Std. diff
Age group						
20-24	0.2	0.3	-0.01	0.3	0.4	-0.02
25-29	0.4	0.6	-0.02	0.5	0.7	-0.03
30-34	1.3	1.6	-0.02	1.3	1.7	-0.03
35-39	4.0	3.6	0.02	4.1	4.4	-0.01
40-44	6.8	7.0	-0.01	6.8	7.4	-0.02
45-49	12.5	11.7	0.03	12.6	12.5	0.00
50-54	18.3	16.2	0.06	18.4	17.1	0.04
55-59	18.1	15.4	0.07	17.8	17.2	0.01
60-64	13.5	13.5	0.00	13.4	14.0	-0.02
65-69	9.5	11.0	-0.05	9.5	9.4	0.00
70-74	7.4	8.8	-0.05	7.3	7.6	-0.01
75-79	5.2	6.2	-0.04	5.2	4.6	0.03
80-84	1.9	3.0	-0.07	1.9	2.0	0.00
85-89	0.7	0.9	-0.02	0.7	0.7	0.00
90-94	<0.2	0.2	-0.03	<0.3	0.2	-0.02
Female	52.8	50.5	0.05	52.5	53.4	-0.02
Charlson comorbidity index*	0.68	0.77	-0.06	0.66	0.69	-0.02
Hyperlipidemia	17.4	15.2	0.06	17.7	17.5	0.00
Hypertensive disorder	24.6	30.1	-0.12	24.5	23.7	0.02
Atrial fibrillation	1.4	1.7	-0.02	1.5	1.4	0.01
Cerebrovascular disease	4.6	3.6	0.05	4.6	4.7	0.00
Peripheral vascular disease	<0.2	0.3	-0.03	<0.3	0.4	-0.04

Heart failure	0.8	1.3	-0.05	0.9	0.9	-0.01
Ischemic heart disease	7.9	15.3	-0.23	7.9	6.7	0.05
Chronic liver disease	0.6	0.4	0.04	0.7	0.7	-0.01
Chronic obstructive lung disease	1.1	1.1	0.00	1.2	0.9	0.03
Renal impairment	0.6	2.0	-0.12	0.7	0.8	-0.02
Gastroesophageal reflux disease	4.6	4.6	0.00	4.7	4.8	-0.01
Osteoarthritis	1.5	2.2	-0.05	1.5	2.0	-0.04
Dementia	0.7	1.0	-0.03	0.8	0.9	-0.02
Depressive disorder	1.4	1.5	0.00	1.5	1.6	-0.01
Visual system disorder	4.3	3.8	0.03	4.2	4.7	-0.02
Medication use						
Beta blocker	17.6	19.8	-0.05	17.6	18.1	-0.01
Calcium channel blockers	15.7	19.6	-0.10	15.7	14.4	0.04
Diuretics	11.7	13.5	-0.05	11.7	10.9	0.03
Antibiotics use	17.6	22.1	-0.11	17.0	16.2	0.02
NSAIDs use	22.4	23.6	-0.03	21.9	21.4	0.01
Drugs for acid related disorders	40.2	43.7	-0.07	39.8	38.7	0.02
Drugs for obstructive airway diseases	4.1	6.1	-0.09	4.1	3.9	0.01
Immunosuppressants	4.1	2.3	0.10	3.8	4.4	-0.03

*Romano's Adaptation of the Charlson comorbidity index was used and presented as mean value. All other variables are presented as percent of the sample size.

PS, propensity score; RAS, renin-angiotensin system; NSAIDs, non-steroidal anti-inflammatory drugs

Table S5. Baseline characteristics of patients with pitavastatin vs. atorvastatin or rosuvastatin in PNUH cohort.

	**Before PS adjustment**			**After PS adjustment**		
	Pitavastatin (n=649)	Atorvastatin+ Rosuvastatin (n=3847)	Std. diff	Pitavastatin (n=585)	Atorvastatin+ Rosuvastatin (n=1048)	Std. diff
Age group						
20-24	<0.8	0.7	-0.03	<0.9	0.6	-0.04
25-29	1.1	0.9	0.02	<0.9	0.8	-0.01
35-39	3.8	2.6	0.07	3.8	4.1	-0.02
40-44	5.0	3.5	0.07	4.6	4.0	0.03
45-49	7.6	5.1	0.10	6.8	6.3	0.02
50-54	12.2	10.3	0.06	12.0	12.4	-0.01
55-59	18.0	15.5	0.07	17.8	19.1	-0.03
60-64	18.1	17.1	0.03	19.0	17.7	0.03
65-69	12.7	14.1	-0.04	12.5	13.2	-0.02
70-74	8.7	12.7	-0.13	9.2	8.1	0.04
75-79	7.5	10.2	-0.10	8.0	7.7	0.01
80-84	2.4	4.3	-0.10	2.6	3.1	-0.03
85-89	<0.8	1.2	-0.07	<0.9	0.9	-0.02
Female	57.8	56.4	0.03	57.9	58.5	-0.01
Charlson comorbidity index*	1.45	1.36	0.04	1.35	1.38	-0.01
Hyperlipidemia	11.0	31.4	-0.52	11.3	9.9	0.04
Hypertensive disorder	16.8	27.3	-0.26	16.6	14.8	0.05
Atrial fibrillation	2.0	3.0	-0.06	2.1	0.7	0.12
Cerebrovascular disease	6.1	9.1	-0.11	6.7	5.6	0.04
Peripheral vascular disease	<0.8	0.4	-0.01	<0.9	<0.5	0.02
Heart failure	1.8	3.8	-0.12	1.9	1.3	0.05
Ischemic heart disease	3.4	11.2	-0.31	3.8	2.8	0.05
Chronic liver	<0.8	1.2	-0.06	<0.9	1.4	-0.07

disease						
Chronic obstructive lung disease	3.0	4.9	-0.10	3.2	2.6	0.04
Renal impairment	2.0	5.3	-0.18	1.9	2.0	-0.01
Gastroesophageal reflux disease	4.3	4.8	-0.03	4.8	4.0	0.04
Osteoarthritis	<0.8	1.5	-0.10	<0.9	1.2	-0.07
Dementia	0.8	1.3	-0.06	0.9	1.0	-0.02
Depressive disorder	2.0	3.3	-0.08	2.2	1.8	0.03
Visual system disorder	6.7	7.1	-0.02	6.7	6.2	0.02
Medication use						
RAS blocker	12.3	18.3	-0.17	12.6	12.1	0.02
Beta blocker	12.5	21.4	-0.24	13.5	11.5	0.06
Calcium channel blockers	8.7	12.4	-0.12	8.7	6.5	0.08
Diuretics	9.6	14.8	-0.16	9.7	8.2	0.05
Antibiotics use	17.2	23.6	-0.16	16.2	14.4	0.05
NSAIDs use	17.5	16.5	0.03	16.2	15.6	0.02
Drugs for acid related disorders	28.5	33.6	-0.11	27.7	25.6	0.05
Drugs for obstructive airway diseases	6.6	7.7	-0.04	6.7	4.9	0.08
Immunosuppressants	5.6	2.1	0.18	4.1	3.8	0.01

*Romano's Adaptation of the Charlson comorbidity index was used and presented as mean value. All other variables are presented as percent of the sample size.

PS, propensity score; RAS, renin-angiotensin system; NSAIDs, non-steroidal anti-inflammatory drugs

Table S6. Baseline characteristics of patients with pitavastatin vs. atorvastatin or rosuvastatin in EUMC cohort.

	Before PS adjustment			After PS adjustment		
	Pitavastatin (n=1159)	Atorvastatin+ Rosuvastatin (n=4570)	Std. diff	Pitavastatin (n=1139)	Atorvastatin+ Rosuvastatin (n=2018)	Std. diff
Age group						
20-24	0.7	0.5	0.02	0.7	0.5	0.02
25-29	<0.4	1.1	-0.12	<0.4	0.4	-0.05
30-34	2.0	2.2	-0.01	1.9	2.5	-0.04
35-39	4.1	3.7	0.02	4.0	4.5	-0.03
40-44	7.5	6.3	0.04	7.5	7.3	0.01
45-49	11.4	10.8	0.02	11.5	12.3	-0.03
50-54	17.7	14.7	0.08	17.7	17.1	0.02
55-59	15.6	15.2	0.01	15.6	15.2	0.01
60-64	13.8	12.8	0.03	13.8	13.1	0.02
65-69	9.8	11.1	-0.04	9.8	9.6	0.01
70-74	9.0	8.8	0.01	9.0	8.3	0.03
75-79	4.9	6.5	-0.07	4.8	4.6	0.01
80-84	2.3	3.8	-0.09	2.3	2.6	-0.02
85-89	0.9	1.9	-0.09	0.9	1.5	-0.06
90-94	<0.4	0.4	-0.04	<0.4	<0.2	0.01
Female	56.0	58.9	-0.06	56.1	54.8	0.03
Charlson comorbidity index*	0.59	0.77	-0.17	0.58	0.61	-0.02
Hyperlipidemia	37.8	33.1	0.10	38.1	41.4	-0.07
Hypertensive disorder	30.4	24.1	0.14	30.3	32.8	-0.06
Atrial fibrillation	0.7	0.5	0.02	0.6	0.6	0.01
Cerebrovascular disease	6.0	7.6	-0.06	6.0	5.7	0.01
Peripheral vascular disease	3.3	3.7	-0.02	3.2	3.2	0.00

Heart failure	0.7	1.3	-0.06	0.6	1.5	-0.09
Ischemic heart disease	11.1	8.4	0.09	11.0	12.3	-0.04
Chronic obstructive lung disease	0.5	0.9	-0.04	0.5	0.6	-0.01
Renal impairment	2.0	1.8	0.02	2.1	1.6	0.04
Gastroesophageal reflux disease	3.1	3.4	-0.02	3.2	3.3	0.00
Dementia	1.0	3.4	-0.16	1.1	1.2	-0.01
Depressive disorder	2.6	2.3	0.02	2.6	1.9	0.05
Visual system disorder	5.0	4.6	0.02	5.0	4.0	0.05
Medication use						
RAS blocker	32.7	28.0	0.10	31.9	35.0	-0.07
Beta blocker	13.9	14.9	-0.03	14.1	14.9	-0.02
Calcium channel blockers	21.9	21.9	0.00	21.8	22.4	-0.01
Diuretics	15.9	13.3	0.07	15.8	17.3	-0.04
Antibiotics use	18.9	22.7	-0.09	18.7	18.0	0.02
NSAIDs use	18.0	23.5	-0.14	17.9	17.5	0.01
Drugs for acid related disorders	30.6	35.8	-0.11	30.4	28.1	0.05
Immunosuppressants	3.0	2.8	0.01	3.1	2.9	0.01

*Romano's Adaptation of the Charlson comorbidity index was used and presented as mean value. All other variables are presented as percent of the sample size.

PS, propensity score; RAS, renin-angiotensin system; NSAIDs, non-steroidal anti-inflammatory drugs

Table S7. Baseline characteristics of patients with pitavastatin vs. atorvastatin or rosuvastatin in NHIMC cohort.

	**Before PS adjustment**			**After PS adjustment**		
	Pitavastatin (n=986)	Atorvastatin+ Rosuvastatin (n=10817)	Std. diff	Pitavastatin (n=865)	Atorvastatin+ Rosuvastatin (n=1637)	Std. diff
Age group						
20-24	<0.5	0.3	0.03	<0.6	0.5	-0.02
25-29	<0.5	0.4	0.00	<0.6	0.7	-0.05
30-34	1.0	0.7	0.03	1.1	0.5	0.07
35-39	1.8	2.4	-0.04	1.9	2.4	-0.03
40-44	4.1	5.6	-0.07	4.0	3.4	0.03
45-49	9.4	8.2	0.04	9.9	9.1	0.02
50-54	14.3	12.2	0.06	14.5	15.4	-0.03
55-59	15.3	13.0	0.07	15.2	13.8	0.04
60-64	14.7	12.1	0.08	14.8	14.7	0.00
65-69	11.4	13.1	-0.05	10.8	11.1	-0.01
70-74	11.7	12.7	-0.03	11.3	10.1	0.04
75-79	8.7	10.1	-0.05	8.9	9.1	-0.01
80-84	4.3	6.0	-0.07	4.5	5.9	-0.06
85-89	2.0	2.7	-0.04	2.0	2.8	-0.05
90-94	<0.5	0.6	-0.06	<0.6	<0.3	0.03
Female	63.8	57.5	0.13	63.3	64.0	-0.01
Charlson comorbidity index*	0.82	0.78	0.03	0.77	0.77	0.00
Hyperlipidemia	19.4	19.1	0.01	21.9	21.8	0.00
Hypertensive disorder	38.7	34.8	0.08	34.6	37.1	-0.05
Atrial fibrillation	1.2	1.7	-0.04	1.4	1.2	0.02
Cerebrovascular disease	6.0	9.4	-0.13	6.8	5.8	0.04
Peripheral vascular disease	6.2	6.0	0.01	5.6	6.4	-0.03
Heart failure	0.6	1.6	-0.10	0.7	1.1	-0.04

Ischemic heart disease	5.7	9.9	-0.16	6.4	6.7	-0.01
Chronic liver disease	<0.5	0.4	-0.07	<0.6	<0.3	-0.01
Chronic obstructive lung disease	1.2	1.4	-0.01	1.2	0.9	0.03
Renal impairment	10.1	3.3	0.27	6.5	8.0	-0.06
Gastroesophageal reflux disease	3.6	2.9	0.04	3.7	2.9	0.05
Osteoarthritis	1.0	1.9	-0.07	1.1	1.5	-0.04
Dementia	2.7	3.7	-0.06	3.2	3.7	-0.03
Depressive disorder	4.0	4.4	-0.02	4.5	3.7	0.04
Visual system disorder	6.2	5.5	0.03	6.5	6.1	0.02
Medication use						
RAS blocker	35.6	29.0	0.14	29.8	34.0	-0.09
Beta blocker	17.8	19.6	-0.05	18.8	20.3	-0.04
Calcium channel blockers	30.4	29.4	0.02	29.2	29.6	-0.01
Diuretics	12.6	15.9	-0.09	13.2	14.9	-0.05
Antibiotics use	15.4	19.6	-0.11	14.7	14.0	0.02
NSAIDs use	19.0	25.8	-0.16	19.7	16.9	0.07
Drugs for acid related disorders	25.0	32.4	-0.16	25.4	23.1	0.05
Drugs for obstructive airway diseases	4.1	6.0	-0.09	3.9	4.0	0.00
Immunosuppressants	0.8	0.8	0.00	0.7	0.8	-0.01

*Romano's Adaptation of the Charlson comorbidity index was used and presented as mean value. All other variables are presented as percent of the sample size.

PS, propensity score; RAS, renin-angiotensin system; NSAIDs, non-steroidal anti-inflammatory drugs

Table S8. Baseline characteristics of patients with pitavastatin vs. atorvastatin or rosuvastatin in MJH cohort.

	Before PS adjustment			After PS adjustment		
	Pitavastatin (n=765)	Atorvastatin+ Rosuvastatin (n=2953)	Std. diff	Pitavastatin (n=695)	Atorvastatin+ Rosuvastatin (n=1173)	Std. diff
Age group						
20-24	<0.6	0.2	0.02	<0.7	<0.4	0.05
25-29	<0.6	0.5	-0.01	<0.7	0.6	-0.02
30-34	1.0	1.1	-0.01	1.2	0.7	0.04
35-39	2.6	3.3	-0.04	2.6	4.4	-0.10
40-44	5.8	5.8	0.00	6.0	6.5	-0.02
45-49	9.7	9.6	0.00	9.6	10.0	-0.01
50-54	15.7	13.6	0.06	15.7	14.1	0.04
55-59	12.6	14.5	-0.06	12.5	14.6	-0.06
60-64	15.5	14.3	0.03	15.7	14.8	0.02
65-69	12.1	10.3	0.06	11.8	10.2	0.05
70-74	9.9	9.7	0.01	9.5	8.3	0.04
75-79	8.1	8.7	-0.02	8.5	7.9	0.02
80-84	4.1	5.3	-0.06	4.5	4.6	-0.01
85-89	1.4	2.3	-0.06	1.0	2.2	-0.10
90-94	<0.6	0.6	-0.01	<0.7	0.8	-0.05
Female	62.5	51.7	0.22	60.6	60.8	0.00
Charlson comorbidity index*	0.69	0.70	-0.01	0.65	0.66	-0.01
Hyperlipidemia	71.3	60.0	0.24	68.5	69.0	-0.01
Hypertensive disorder	37.2	40.9	-0.07	37.7	40.4	-0.06
Atrial fibrillation	1.2	1.9	-0.06	1.3	0.9	0.03
Cerebrovascular disease	6.7	8.5	-0.07	6.9	7.7	-0.03
Peripheral vascular disease	1.5	0.9	0.06	1.4	1.2	0.03
Heart failure	6.4	7.2	-0.03	6.3	5.3	0.05

Ischemic heart disease	7.7	18.6	-0.33	8.3	6.1	0.09
Chronic liver disease	<0.6	0.5	-0.04	<0.7	0.4	-0.02
Chronic obstructive lung disease	<0.6	1.0	-0.10	<0.7	1.1	-0.10
Renal impairment	3.0	1.3	0.12	2.7	1.4	0.10
Gastroesophageal reflux disease	11.9	18.4	-0.18	12.2	12.7	-0.01
Osteoarthritis	3.0	3.0	0.00	3.0	2.8	0.01
Dementia	3.6	4.9	-0.06	3.9	5.0	-0.05
Depressive disorder	6.2	6.0	0.01	5.6	5.9	-0.01
Schizophrenia	1.2	0.4	0.08	1.3	0.4	0.09
Visual system disorder	5.2	6.4	-0.05	5.0	7.0	-0.08
Medication use						
RAS blocker	25.8	27.2	-0.03	26.2	26.8	-0.01
Beta blocker	23.8	24.2	-0.01	24.0	21.4	0.06
Calcium channel blockers	9.0	10.6	-0.05	9.8	7.7	0.07
Diuretics	15.2	14.9	0.01	15.7	15.8	0.00
Antibiotics use	23.6	27.8	-0.10	24.5	22.2	0.05
NSAIDs	24.2	28.1	-0.09	25.2	25.0	0.00
Drugs for acid related disorders	40.2	47.9	-0.16	40.4	37.9	0.05
Drugs for obstructive airway diseases	4.8	4.4	0.02	4.7	4.0	0.04
Immunosuppressants	0.6	0.9	-0.03	0.7	1.6	-0.08

*Romano's Adaptation of the Charlson comorbidity index was used and presented as mean value. All other variables are presented as percent of the sample size.

PS, propensity score; RAS, renin-angiotensin system; NSAIDs, non-steroidal anti-inflammatory drugs

Table S9. Baseline characteristics of patients with pitavastatin vs. atorvastatin or rosuvastatin in KWMC cohort.

	**Before PS adjustment**			**After PS adjustment**		
	Pitavastatin (n=522)	Atorvastatin+ Rosuvastatin (n=8178)	Std. diff	Pitavastatin (n=506)	Atorvastatin+ Rosuvastatin (n=987)	Std. diff
Age group						
25-29	<0.9	0.5	-0.01	<1.0	<0.5	0.02
30-34	<0.9	0.9	-0.02	<1.0	1.0	-0.02
35-39	1.1	1.7	-0.04	1.2	2.1	-0.07
40-44	3.2	3.6	-0.02	3.4	3.9	-0.03
45-49	5.8	5.4	0.02	5.9	6.3	-0.02
50-54	10.0	9.7	0.01	10.1	9.1	0.03
55-59	14.1	12.3	0.06	14.8	13.1	0.05
60-64	12.8	13.2	-0.01	13.4	14.3	-0.03
65-69	13.9	12.4	0.04	13.6	12.6	0.03
70-74	14.3	13.7	0.02	13.8	12.2	0.05
75-79	11.7	12.5	-0.03	11.3	13.0	-0.05
80-84	7.5	8.8	-0.05	6.9	7.8	-0.03
90-94	0.9	1.3	-0.03	1.0	1.3	-0.03
Female	57.8	51.9	0.12	57.9	57.0	0.02
Charlson comorbidity index*	0.64	0.76	-0.11	0.65	0.68	-0.03
Hyperlipidemia	42.7	40.8	0.04	44.3	43.0	0.03
Hypertensive disorder	27.9	22.3	0.13	28.1	32.0	-0.09
Atrial fibrillation	5.8	3.7	0.10	5.9	6.1	-0.01
Cerebrovascular disease	6.0	11.7	-0.20	6.1	5.6	0.02
Peripheral vascular disease	1.3	1.1	0.02	1.4	0.7	0.07
Heart failure	3.4	4.0	-0.03	3.6	4.9	-0.07
Ischemic heart disease	16.0	16.3	-0.01	15.0	17.1	-0.06

Chronic liver disease	<0.9	0.3	0.06	<1.0	<0.5	0.04
Chronic obstructive lung disease	2.3	2.1	0.01	2.4	1.8	0.04
Renal impairment	0.9	1.8	-0.08	1.0	1.5	-0.04
Gastroesophageal reflux disease	4.0	3.6	0.02	3.8	5.3	-0.08
Osteoarthritis	2.3	2.1	0.01	2.4	2.5	-0.01
Dementia	1.9	2.7	-0.06	2.0	2.7	-0.05
Depressive disorder	4.3	3.5	0.04	4.0	3.8	0.01
Visual system disorder	6.6	8.1	-0.06	6.3	7.7	-0.05
Medication use						
RAS blocker	35.4	26.4	0.20	34.0	37.2	-0.07
Beta blocker	20.9	21.2	-0.01	21.1	22.6	-0.04
Calcium channel blockers	10.2	9.1	0.04	10.3	11.4	-0.04
Diuretics	21.7	14.8	0.18	19.8	23.1	-0.08
Antibiotics use	23.9	23.4	0.01	23.5	25.4	-0.04
NSAIDs	20.2	19.1	0.03	20.2	20.0	0.00
Drugs for acid related disorders	42.0	43.7	-0.03	42.1	41.1	0.02
Drugs for obstructive airway diseases	5.1	4.9	0.01	5.1	3.7	0.07
Immunosuppressants	<0.9	0.5	0.01	<1.0	0.7	-0.04

*Romano's Adaptation of the Charlson comorbidity index was used and presented as mean value. All other variables are presented as percent of the sample size.

PS, propensity score; RAS, renin-angiotensin system; NSAIDs, non-steroidal anti-inflammatory drugs
